# Phenolics from *Barleria cristata* var. Alba as carcinogenesis blockers against menadione cytotoxicity through induction and protection of quinone reductase

**DOI:** 10.1186/s12906-018-2214-9

**Published:** 2018-05-22

**Authors:** Ali M. El-Halawany, Hossam M. Abdallah, Ahmed R. Hamed, Hany Ezzat Khalil, Ameen M. Almohammadi

**Affiliations:** 1 Department of Natural Products, Faculty of Pharmacy, King Abdulaziz University, Jeddah, 21589 Saudi Arabia; 2 Department of Pharmacognosy, Faculty of Pharmacy, Cairo University, Cairo, 11562 Egypt; 3 Phytochemistry Department and Biology Unit lab 610, Central Laboratory for the Pharmaceutical and Dug Industries Research Division, National Research Centre, Giza, Dokki 12622 Egypt; 4 Department of Pharmacognosy, Faculty of Pharmacy, Minia University, Minia, 61519 Egypt; 5 Department of Pharmaceutical Sciences, College of Clinical Pharmacy, King Faisal University, Al-Ahsa, Saudi Arabia; 6 Department of Clinical Pharmacy, Faculty of Pharmacy, King Abdulaziz University, Jeddah, 21589 Saudi Arabia

**Keywords:** Chemoprevention, Quinonereductase 1, Menadione, Phenolic compounds

## Abstract

**Background:**

There are increasing interests in natural compounds for cancer chemoprevention. Blocking agents represent an important class of chemopreventive compounds. They prevent carcinogens from undergoing metabolic activation and thereby suppressing their interaction with cellular macromolecular targets.

**Methods:**

The effect of phenolic compounds isolated from *Barleria cristata* var. alba as chemopreventive agent was evaluated. The ethyl acetate fraction of *B. cristata* was subjected to different chromatographic techniques for isolation of its major phenolic compounds. The isolated compounds were evaluated for their potential to induce the cancer chemopreventive enzyme marker NAD(P)H quinonereductase 1 (NQO1) in murine Hepa-1c1c7 cell model.

**Results:**

The ethyl acetate fraction of *B. cristata* var. alba yielded five known compounds identified as verbascoside (**1),** isoverbascoside (**2),** dimethoxyverbascoside (**3)**, *p*-hydroxy benzoic acid (**4)**, and apigenin-7-O-glucoside (**5).** Among the tested compounds, isoverbascoside (**2**) was shown to potently induce the activity of the enzyme in a dose –dependent manner. As a functional assay for detoxification, compound **2** was the strongest to protect Hepa-1c1c7 against the toxicity of menadione, a quinone substrate for NQO1.

**Conclusion:**

This effect seemed to be attributed to the compound’s potential to induce both the catalytic activity and protein expression of NQO1 as revealed by enzyme assay and Western blotting, respectively.

**Electronic supplementary material:**

The online version of this article (10.1186/s12906-018-2214-9) contains supplementary material, which is available to authorized users.

## Background

Cancer is a condition in which a cell divides and grows in an uncontrolled manner forming a neoplastic tumor which may spread to other tissues and becomes malignant. Cancer incidence is neither rare anywhere in the world, nor restricted to rich countries. However, deaths from cancer in developing countries are higher than in developed countries [1]. Therefore it is becoming highly recognized that the strategies for cancer prevention are forming the logic and cost effective approach to control this global problem of cancer mortalities as alternative to the high costs of the chemotherapy or radiotherapeutic programs and this should reflect positively on the local and global economies. There are increasing interests in natural compounds for chemoprevention against cancer. These great interests are mainly due to a high number of population studies that showed reduced cancer risk in people consuming dietary phytochemicals compared to people who consume less dietary phytochemicals [2]. Based on the classified stages of carcinogenesis process, the term ‘cancer chemoprevention’ has been defined as the use of relatively non-toxic chemical agent (natural or synthetic) to inhibit, arrest or reverse the carcinogenesis at early stages [3, 4]. Blocking agents is one important class of compounds in chemoprevention. They prevent carcinogens from undergoing metabolic activation and thereby suppressing their interaction with cellular macromolecular targets such as DNA, RNA and proteins. Blocking agents elicit chemopreventive actions through the induction of a set of phase II detoxifying and antioxidant enzymes such as the chemopreventive marker NAD(P)H quinonereductase 1 (NQO1 or QR1), glutathione-s-transferase (GST), UDP-glucuronosyltransferase (UGT), γ-glutamate cysteine ligase (γ -GCL), glutathione reductase (GR), catalase and Mn superoxide dismutase which act in the detoxification and elimination of harmful reactive intermediates and oxidative stress [5]. There have been accumulating evidences for the promising value of natural products in chemoprevention against cancer. The reason for this is largely due to their low toxicity and high diversity of their chemical structures [6].

Genus *Barleria* is belonging to family Acanthaceae. Ten species from this genus are growing in Saudi flora. Plants belonging to this genus are widely used as an ethnomedicines for variety of illnesses [7, 8]. *Barleria cristata* L. as an example of this genus is an ornamental plant with two varieties named on the basis of their flower color including var. alba and var. purpurea for the white and purple flowers, respectively. *B. cristata* var. purpurea is also known as Philippine Violet or Blue Bell Barleria. Leaves and stems of the plant are used as anti-inflammatory, anemia treatment, antiplasmodial, anti-oxidant and analgesic for toothache. The plant was reported to contain alkaloids, flavonoids, iridoids and to be rich in phenolic contents [8].

The phenolic content of the plant encouraged its use as a chemopreventive and cytoprotective agent. In addition, there is no published chemical or biological data on the other variety (alba) of the plant, which encouraged us to investigate its possible chemopreventive effect, based on expected similarity in active constituents’ classes to that of the variety purpurea. Therefore, in continuation to our interest in isolating bioactive chemopreventive agent from plants [5] and based on the chemopreventive activity of phenolics [9], which represent a major constituent in, *B.cristata* var. alba, the current study was performed.

## Methods

### General experimental procedures

UV spectra were recorded in MeOH using a UV IKON940 spectrophotometer.1D and 2D NMR spectra (chemical shifts in ppm and coupling constants in Hz) were recorded on a Bruker DRX-600 MHz Ultrashield spectrometer (Bruker BioSpin, Billerica, MA, USA) using DMSO-d6 as solvent, with TMS as the internal reference. Column chromatographic separations were performed on silica gel 60 (70–230 mesh, Merck, Darmstadt, Germany), Silica gel 100C_18_-Reversed phase (0.04–0.063 mm, Merck, Darmstadt, Germany) and Sephadex LH-20(Pharmacia Fine Chemicals Inc., Uppsala, Sweden). TLC analysis was performed on pre-coated TLC plates with silica gel 60 F_254_(Merck, Darmstadt, Germany). Purification of the isolated compounds was performed on a preparative HPLC Agilent1200 equipped with a multi-wavelength detector and ZorbaxSB-C18 column (9.4 × 250 mm).

### Plant material

Aerial parts of *B. cristata* L. var. alba were collected from El-Orman Public Garden, Giza, Egypt during April and June 2010. Authentication of plant material was established by Agricultural Engineer Traiz Labib, taxonomy specialist in El-Orman public garden, Giza, Egypt. Voucher specimen (Reg. No. BS1110) is kept in the Herbarium of the Department of Natural Products and Alternative Medicine, Faculty of Pharmacy, King Abdulaziz University, KSA.

### Materials for the biology

All chemicals and reagents for cell culture and bioassays were purchased from Lonza (Verviers, Belgium) or Sigma-Aldrich (Steinheim, Germany). Plastic ware for cell culture and assays were prom Griener Bio One (Frickenhausen, Germany). Murine hepatoma cells (Hepa1c1c7, ATCC, USA).

### Extraction and isolation of main active constituents

The air dried powdered aerial parts (3 Kg) were exhaustively extracted at room temperature (14 days) using 25 L 70% methyl alcohol, applying cold maceration method to avoid damage of active principals. The solvent mixtures were distilled off under reduced pressure using rotary evaporator and then freeze-dried to yield the total dry extract (210 g), which was stored in deep freezer for further analysis. The total extract was suspended in water (500 mL) and fractionated with chloroform (3 × 1 L) followed by EtOAc (3 × 1 L) and finally *n*-butanol saturated with water. The EtOAc fraction (10 g) was fractionated using RP-18 VLC (5 × 20 cm) using MeOH:H_2_O (0–70% *v*/v, 5% increment) and four major fractions were collected. Fraction 1 (2.3 g) was placed on silica gel column (2 × 50 cm) using CHCl_3_:MeOH (9.5:0.5 *v*/v) as mobile phase. Subfraction 1–5 was further purified using Sephadex LH-20 (2 × 50 cm) using MeOH as an eluent to give 50 mg of **1**. Fraction 2 was re-chromatographed on silica gel column (2 × 50 cm) using Hexane:EtOAc (1:1) to give 20 mg of compound **2**.Subfraction3 was purified on RP-18 HPLC using the following mobile phase system acetonitrile: water (2:8) to yield two pure compounds **3** (3 mg) and **4** (5 mg). Fraction 4 was purified on RP-18 HPLC using the following mobile phase system acetonitrile: water (3:7) to yield compound **5** (2 mg).

### Hepa-1c1c7 cell culture

Murine hepatoma cell line Hepa-1c1c7 was maintained as monolayer culture in α- modified Minimum Essential Medium Eagle (α-MEME) supplemented with 10% (*v*/v) heat-and charcoal–inactivated fetal bovine serum, 2 mM L-glutamine, 100 U/ml penicillin, 100 μg/ml streptomycin sulphate in humidified incubator (Sartorius CMAT, Germany, 5% CO_2_/95% air). At about 80% confluence, cells were routinely sub-cultured with Trypsin EDTA solution.

### Assessment of the induction of NQO1 in Hepa-1c1c7 cells

The induction of NQO1 in Hepa-1C1C7 cells was assessed. Briefly, cells (1.5 × 10^5^ cells/ml) were seeded onto 6-well plates and left overnight to adhere and form semi-confluent monolayers. Monolayers were treated with either vehicle (final concentration 0.5% *v*/v DMSO) or test compounds for additional 48 h. Media were then aspirated and monolayers were washed with ice-cold Dulbecco’s PBS (1 ml/well). Cells were then scrapped in ice-cold lysis buffer (25 mMTris-Cl, pH 7.4, 250 mM sucrose and 5 μM FAD) and transferred to microcentrifuge tubes. Cell suspensions were then sonicated on ice for 5 s (20% amplitude). Sonicates were then centrifuged (15,000×g for 10 min) and the supernatant (cytosolic fraction) was aliquoted and stored at − 80 °C until assayed.

### NQO1 assay

The dicoumarol-sensitive NQO1 activity was measured in cell lysate according to [10] as optimized in our laboratory for spectrophotometer measurement in 500 μl cuvettes. Briefly, the reaction mixture contained in a final volume of 500 μl: 25 mM Tris buffer (pH 7.4), 0.7 mg/ml bovine serum albumin, 5 μM FAD, 0.2 mM β-NADH as electron donor, 20 μM 2,6-dichlorophenolindophenol (DCPIP) as electron acceptor in the absence (total reductase activity) or the presence of 20 μM dicoumarol (inhibited non-NQO-1 activity). The reaction was started by the addition of 10 μl of supernatant of cell lysates to 490 μl of the reaction mixture in disposable cuvettes. The kinetic determination of enzyme activity was carried out using UV-visible spectrophotometer monitoring the decrease in absorbance of DCPIP at 600 nm for 1 min. The instrument software was used to calculate the average reaction rate per minute (ΔA/minute). Total proteins were determined using Bradford assay [11]. The enzyme activity was normalised to 1 mg of total proteins and expressed as ΔA/minute/mg protein.

### Effect of compounds on menadione-induced cytotoxicity in Hepa-1c1c7 cells

First, The cytotoxic effect of menadione (MD), a quinone substrate for NQO1, on Hepa-1c1c7 cells was determined using a modified sulforhodamin B assay for cellular protein content which is essentially based on method mentioned by [12]. Briefly, Hepa-1c1c7 cells (100,000 cells/ml) were seeded onto 96-well plates and incubated to adhere overnight at 5% CO_2_/95% air incubator. At the next day, cells were treated with either vehicle (0.1% DMSO) or increasing concentrations of menadione (1.5–100 μM) and incubated for further 24 h. Culture medium was then aspirated and monolayers were fixed with 10% TCA for 1 h at 4 °C after which all wells were washed with deionized water (4 washes). Air-dried plates were then stained with 0.4% SRB in 1% acetic acid for 30 min at room temperature. Excess dye was removed with washing with 1% acetic acid (4 washes). Bound SRB in air-dried plates were then quantified by solubilisation in 10 mM unbuffered Tris base. The absorbance (OD) was read at 540 nm on a microplate reader (FLUOstar Optima, BMG LABTECH GmbH, Ortenberg, Germany). Percentage viability to vehicle control (100%) was calculated from the mean values of the OD of test concentrations. Dose response curves were plotted on Graphpad Prism V6.0 and analysed using non-linear regression to calculate the concentration of MD causing 50% cytotoxicity (IC_50_).

To test the potential of compounds **1**, **2**, **4** and **5** to protect Hepa-1c1c7 cells against MD cytotoxicity, cells were pre-treated with either DMSO or 3.125 μM from compounds for 24 h before being intoxicated with 20 μM of menadione (MD) for a further 24 h. The Plate was then air-dried and processed for SRB cytotoxicity assay as mentioned above.

### NQO1 western blotting

Cells were cultured and treated as mentioned above. NQO1 protein expression was assessed in cell sonicates by Western blotting. Samples, including vehicle control, positive control (sulforaphane at 5 μM) and compound **2**-treated lysates (15 μg total proteins/lane) were resolved by electrophoresis on 10% acrylamide/bis acrylamide gel (200 Volts for 1 h). Resolved proteins were then transferred to nitrocellulose membrane at 100 V for 90 min. Membranes were blocked in 5% non-fat milk in Tris-buffered saline with 0.1% Tween 20 (TBST) for 1 h at 25 °C and then probed overnight (4 °C) with primary antibodies against NQO1 and β-actin (abcam, UK). After three washes in TBST (10 min each), membranes were probed with appropriate secondary antibodies for 1 h at 25 °C, washed three times in TBST and then developed using enzyme chemiluminescence (ECL). Protein bands were visualized on developed membranes using x-ray film. Protein band intensities were determined and normalized to β-actin using Image Studio Lite software v 5.2 (Li-COR® Biosciences, USA).

## Results

The ethyl acetate fraction of *B. cristata* var. alba yielded five known compounds. The isolated compounds (Fig. 1) were identified based on their NMR data (Additional file 1: Figures S1–10) and by comparison with the reported data as; verbascoside (**1**) [13], isoverbascoside (**2**) [13], dimethoxyverbascoside (**3**), p-hydroxybenzoic acid (**4**) [14] and apigenin-7-O-glucoside (**5**) [15]*.*

**Fig. 1:**
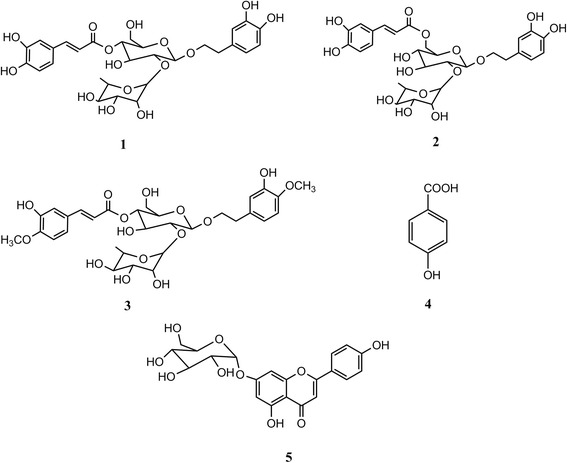
Chemical structure of isolated phenolic constituents from *B. cristata*varalba

### Induction of NQO1 activity by B.Cristata var. alba phenolic constituents

**Fig. 2:**
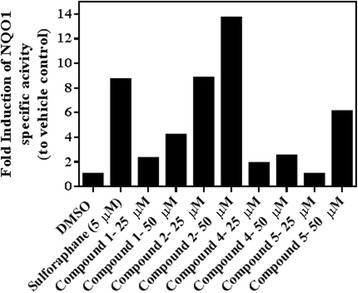
Fold induction of NQO1 activity by isolated compounds **1**,**2**,**4** and **5**.Hepa-1c1c7 were treated with either vehicle (DMSO) or indicated concentrations of the compounds for 48 h and then cell lysates were assayed for NQO1 activity as described in the *Materials and Methods* section

In the present study, we evaluated compounds (**1**, **2**, **4** & **5**) isolated from *B. cristata* for their potential to induce the cancer chemopreventive marker enzyme NQO1 in murine Hepa-1c1c7 cells. A shown in Fig. 2, tested compounds caused differential potencies as inducers for the activity of NQO1 following a 48 h treatment period. The most active compound was shown to be compound **2** (isoverbascoside) causing 8.8-fold induction of NQO1 activity at 25 μM over vehicle control activity level. Higher inducer potency (13.9-fold over control) was recorded with 50 μM of compound 2. Lower inducer activities were shown by compounds **1**, **4** and **5** as displayed in Fig. 2.

### Effect of compounds from *B. cristata* on menadione-induced toxicity (functional assay of NQO1)

**Fig. 3:**
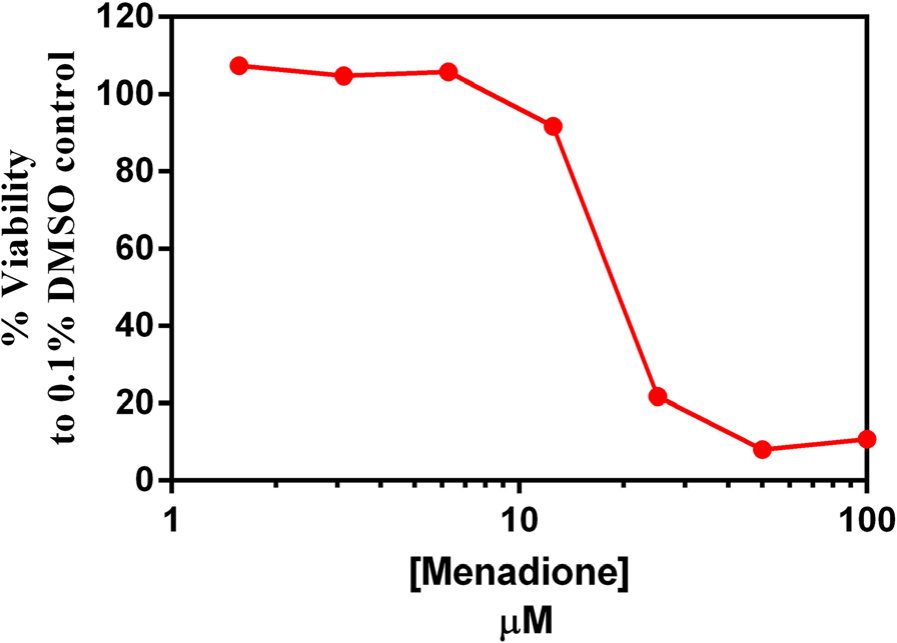
Concentration-response curve of the toxicity of Menadione on Hepa-1c1c7 cells. Cells were cultured and treated with MD (1.5–100 μM) for 24 h and then assayed using SRB cytotoxicity assay as described in the Experimental section, each data point represents means of Octuplet well treatments

**Fig. 4:**
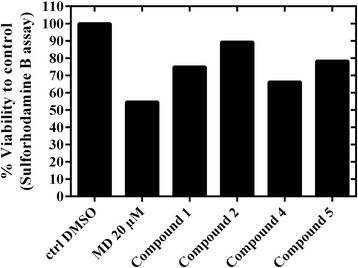
Protection of Hepa-1c1c7 cells against menadione-induced cytotoxicity**.** Hepa-1c1c7 cells were pretreated with either DMSO or 3.125 μM from indicated compounds for 24 h before being intoxicated with 20 μM of menadione (MD) for a further 24 h. Cell monolayers were then fixed with TCA for 1 h and processed for SRB cytotoxicity assay. Data are means of at least quadrate treatments for each concentration

Among the tested compounds (Fig. 3), compound **2** (isoverbascoside) was the most active one to protect Hepa-1c1c7 cells against MD cytotoxicity. This is matched with the potency of this compound as NQO1 inducer (Fig. 2). The induction of NQO1 by isoverbascoside helped to detoxify MD through 2-electron reduction to the hydroquinone form menadiol. Menadiol is easily excreted and this prevents the phase I single electron reduction of MD to the toxic semiquinone. Lower protection was produced by the other compounds which can be ordered from high to low activity as Compound 5 > Compound 1 > Compound 4 as displayed in Fig. 4.

**Fig. 5:**
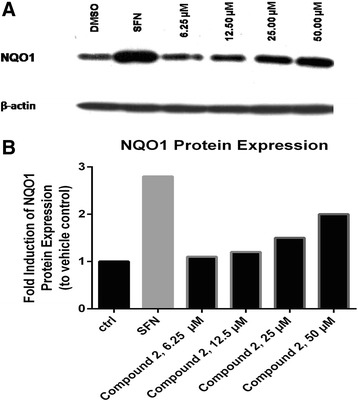
Concentration -dependent upregulation of NQO1 protein expression by Compound 2 in Hepa1c1c7 cells. Cells were cultured and assayed using Western blotting (**a**) as described in the Experimental section. Densitometric determination (**b**) of the fold of NQO1 induction over DMSO control (ctrl) was performed using Image Studio Lite

Compound **2** was also shown to cause a concentration-dependent upregulation of the protein expression level of NQO1 using Western blotting analysis as displayed in Fig. 5a and b.

Taken together, the present study shed some light on the mechanism-based potential of the phenolic constituents of the ornamental plant *B. cristata* var. alba as chemopreventive agents. As shown, this activity is through the induction of the cytoprotective enzyme NQO1 at the activity and protein expression levels.

## Discussion

Many experimental evidences exist to support the protective roles of NQO1 in the prevention against the toxicity and neoplastic effects of quinones, quinoneimenes and azo dyes in vitro as well as in experimental animals. For example, Wu et al., [16] investigated the protective role of indole-3-carbinol (I3C), a known cruciferous NQO1 inducer, to reduce the incidence of prostate cancer in mice model of transgenic adenocarcinoma of mouse prostate (TRAMP mice). Feeding TRAMP mice with 1% I3C diet has significantly abolished the number of palable tumors, compared with the untreated TRAMP group. This effect was found to occur via the induction of the Nrf2-target gene NQO1 expression. The induction of Nrf2-target genes NQO1 and Glutathione S Transferase (GST) by ursolic acid inhibited the development of lung cancer in nude mice injected with the lung cancer cells A549 [17]. Moreover, inducers of NQO1 in vitro in the Hepa-1c1c7 model were also able to suppress the carcinogenic effects of diverse cancer promoting agents related to different types of cancers, with sulforaphane being the prototype NQO1 inducer [18, 19]. Phenolic phytochemicals were found to be potent inducers of NQO1 by many investigators. For example, (+)-tephropurpurin, a chalcone isolated from *Tephrosia purpurea* induced NQO1 activity in the murine hepatoma hepa-1c1c7 model [20]. Protection against azoxymethane-induced intestinal adenocarcinoma was accomplished in F344 rats administered with the flavonoid morin via significant increase in the activity of GST and NQO1 enzymes over those of untreated control [21]. Morin was also shown to protect against 4-nitroquinone 1-oxide-induced tongue carcinogenesis in F344 rat through induction of GST and NQO1 enzymatic activities [22].

In present study; to further elucidate the potency of tested compounds as inducers of the cancer chemopreventive marker NQO1 at the functional level, we used an assay system at which the NQO1 substrate menadione (MD), a toxic quinone which is known to cause redox cycling and oxidative damage through 1-electron reduction catalyzed by NADH Cytochrome P450 reductase and this results in the formation of unstable toxic semiquinone [23]. NQO1 is known to detoxify quinones through direct 2-electron-reduction into the more water soluble, more stable, easily conjugated hydroquinone and therefore minimizing the formation of the toxic semiquinone metabolite of MD [13, 24].

Treatment of Hepa-1c1c7 cells with 20 μM MD alone caused a dramatic loss of cell viability near to 50% compared to DMSO control (calculated MD IC_50_ from dose response curve = 19.7 μM, Fig. 3). Pretreatment of Hepa-1c1c7 cells with 3.125 μM from tested compounds inhibited the cytotoxic effect caused by MD treatment, displaying higher cell viability % of cells compared to the 20 μM MD only treated cells. Therefore, the protection of Hepa-1c1c7 cells pretreated by compounds **1**, **2**, **4** or **5**, seems to be largely attributed to their NQO1 inducer activities (Fig. 2) that resulted in detoxification of MD compared to MD treated cells.

## Conclusion

The present study shed light on the mechanism-based potential of the phenolic constituents of the ornamental plant *B. cristata* var. alba as chemopreventive agents. As shown, this activity is through the induction of the cytoprotective enzyme NQO1 at the activity and protein expression levels.

## Additional file

Additional file 1: Supplementary data contains ten supplementary figures showing the H and 13C-NMR data of isolated compounds 1–5. (PDF 1541 kb)
