# Supplementary material for: Phenolics from Barleria cristata var. Alba as carcinogenesis blockers against menadione cytotoxicity through induction and protection of quinone reductase
Source: BMC Complement Altern Med. 2018 May 22;18:163. doi: 10.1186/s12906-018-2214-9 (PMC5964735; doi:10.1186/s12906-018-2214-9)
Supplement: Supplementary file 1 — Supplementary data contains ten supplementary figures showing the H and 13C-NMR data of isolated compounds 1–5. (PDF 1541 kb) [file 12906_2018_2214_MOESM1_ESM.pdf]

Phenolics from *Barleria cristata* var. alba as Carcinogenesis Blockers; Emphasis on Quinone Reductase Induction and Protection Against Menadione Cytotoxicity

Ali M. El-Halawany[1,2], Hossam M. Abdallah[1,2,*], Ahmed R.Hamed[3], Hany Ezzat Khalil[4, 5], Ameen M. Al-Mohammadi [6]

[1]*Department of Pharmacognosy, Faculty of Pharmacy, Cairo University, Cairo 11562, Egypt*

[2]*Department of Natural Products, Faculty of Pharmacy, King Abdulaziz University, Jeddah 21589, Saudi Arabia*

[3]*Phytochemistry Department, Pharmaceutical and Dug Industries Research Division, National Research Centre, Dokki, Giza, 12622, Egypt.*

[4]*Department of Pharmacognosy, Faculty of Pharmacy, Minia University; Minia, 61519, Egypt.*
[5]*Department of Pharmaceutical Sciences, College of Clinical Pharmacy, King Faisal University, Al-Ahsa, Saudi Arabia.*
[6]*Department of Clinical Pharmacy, Faculty of Pharmacy, King Abdulaziz University, Jeddah 21589, Saudi Arabia*

*Corresponding to: Hossam M. Abdallah, Department of Natural Products, Faculty of Pharmacy, King Abdulaziz University, Jeddah 21589, Saudi Arabia

E-mail address: hmafifi2013@gmail.com

Supporting information

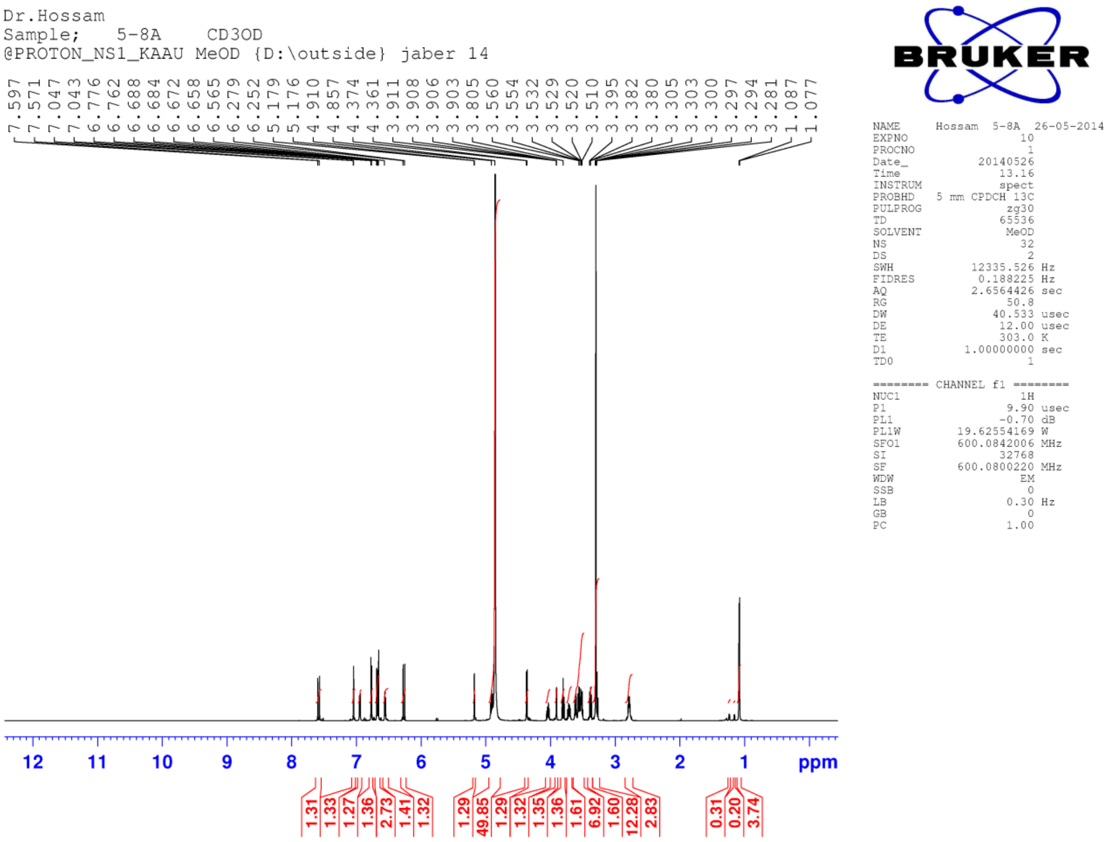

Supplementary Figure 1: HNMR of compound 1

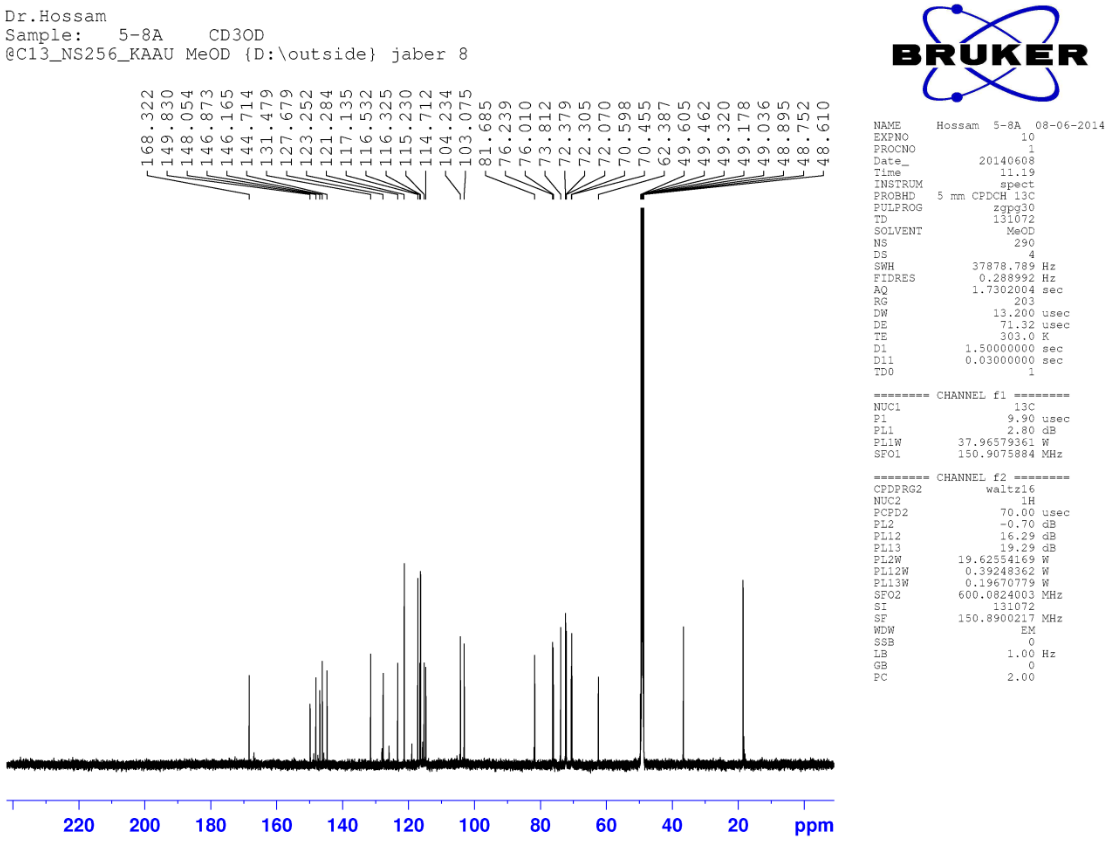

Supplementary Figure 2: ^{13}CNMR of compound 1

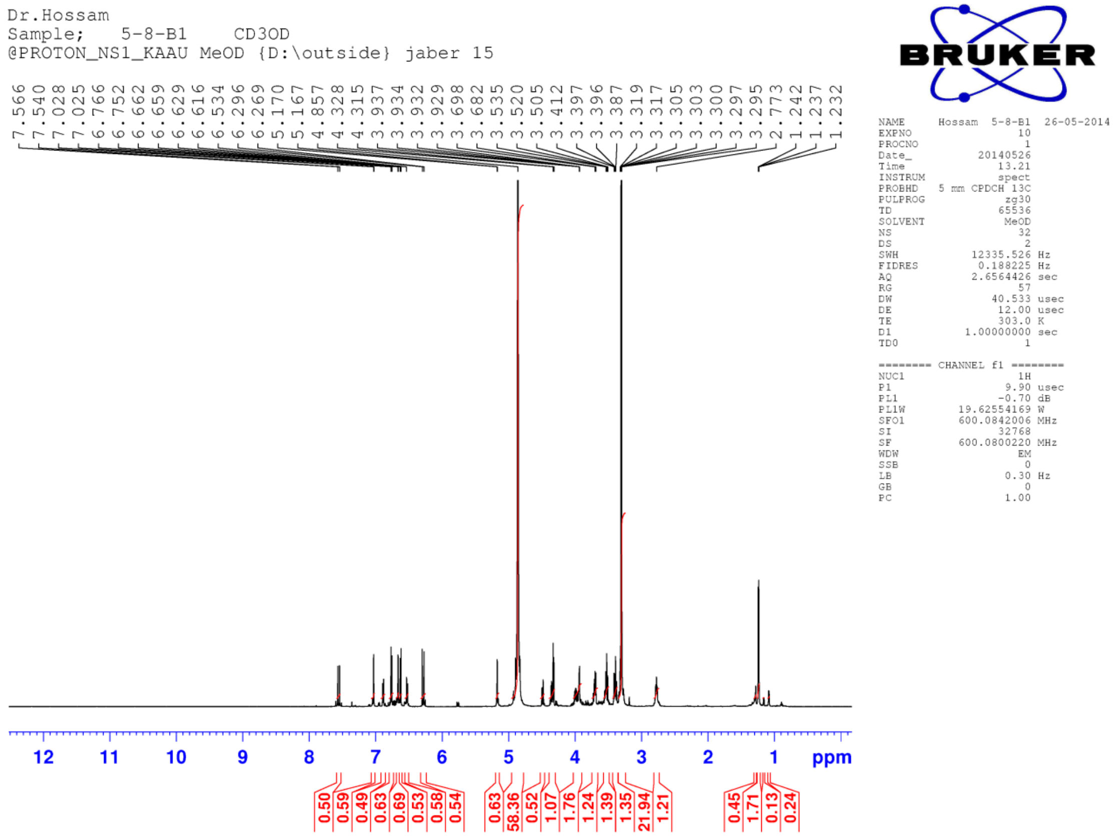

Supplementary Figure 3: HNMR of compound 2

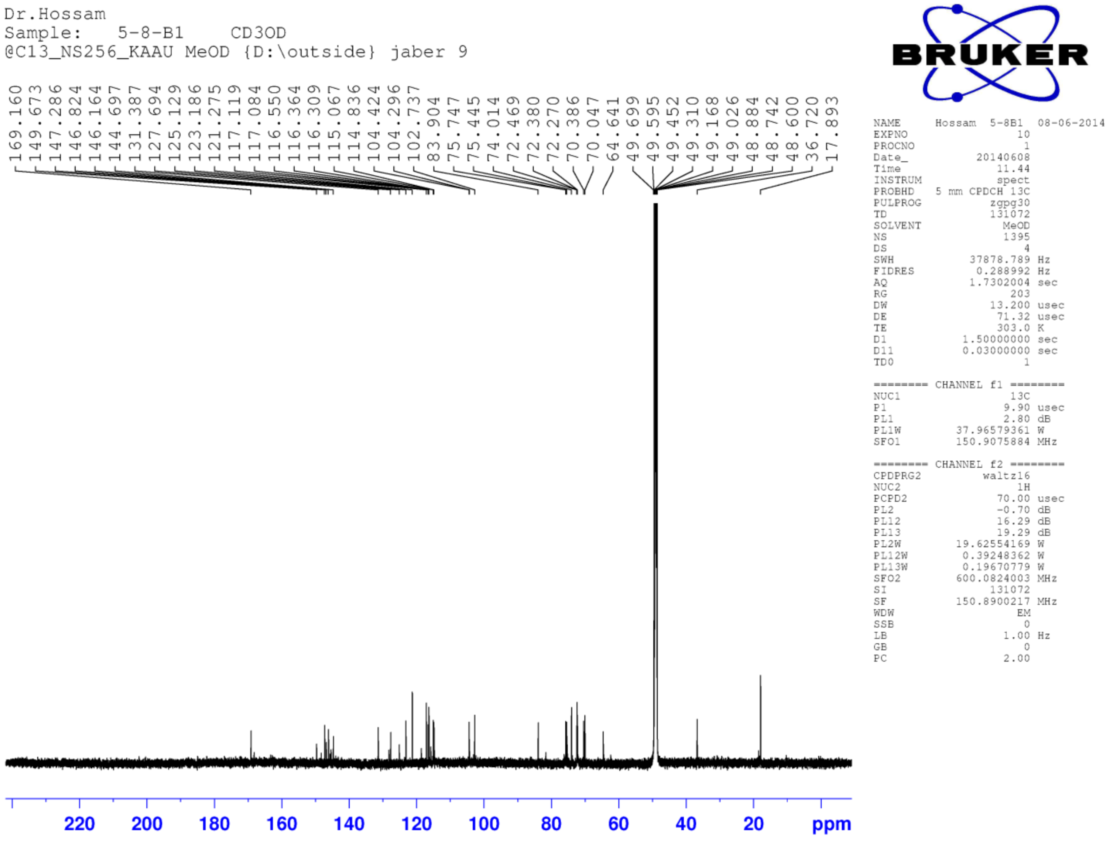

Supplementary Figure 4: ^{13}CNMR of compound 2

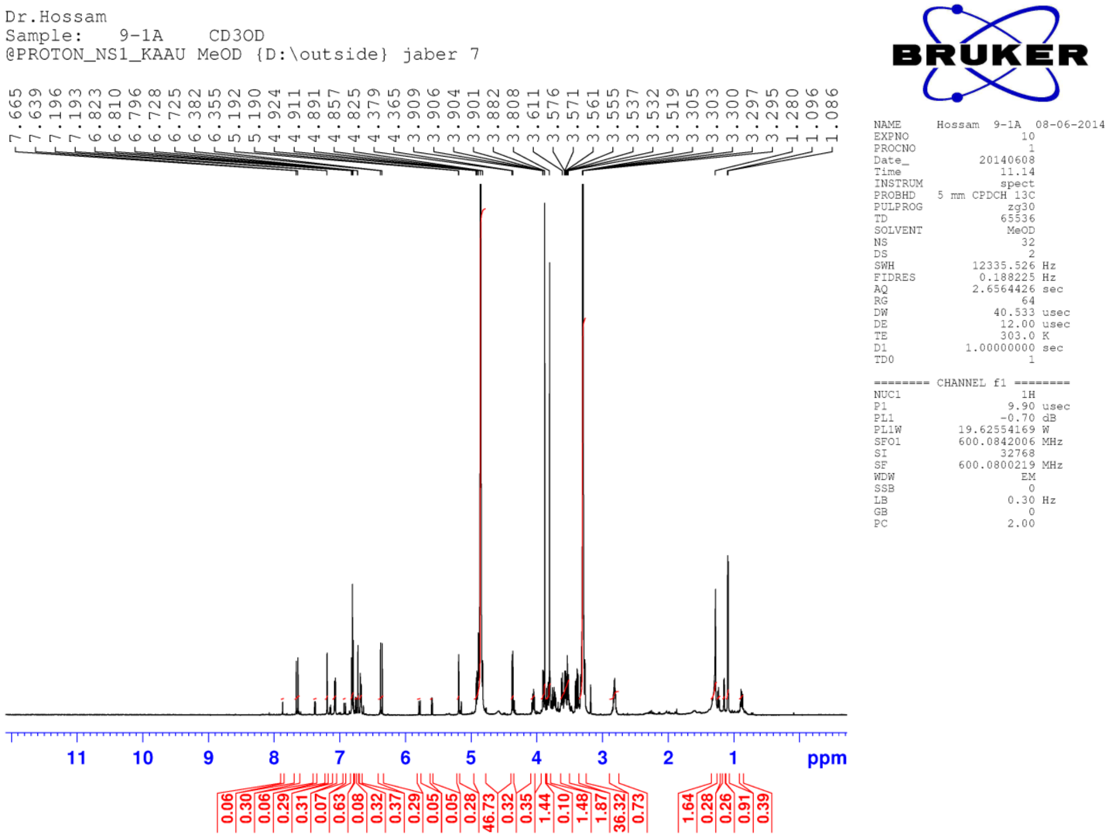

Supplementary Figure 5: ^{13}CNMR of compound 3

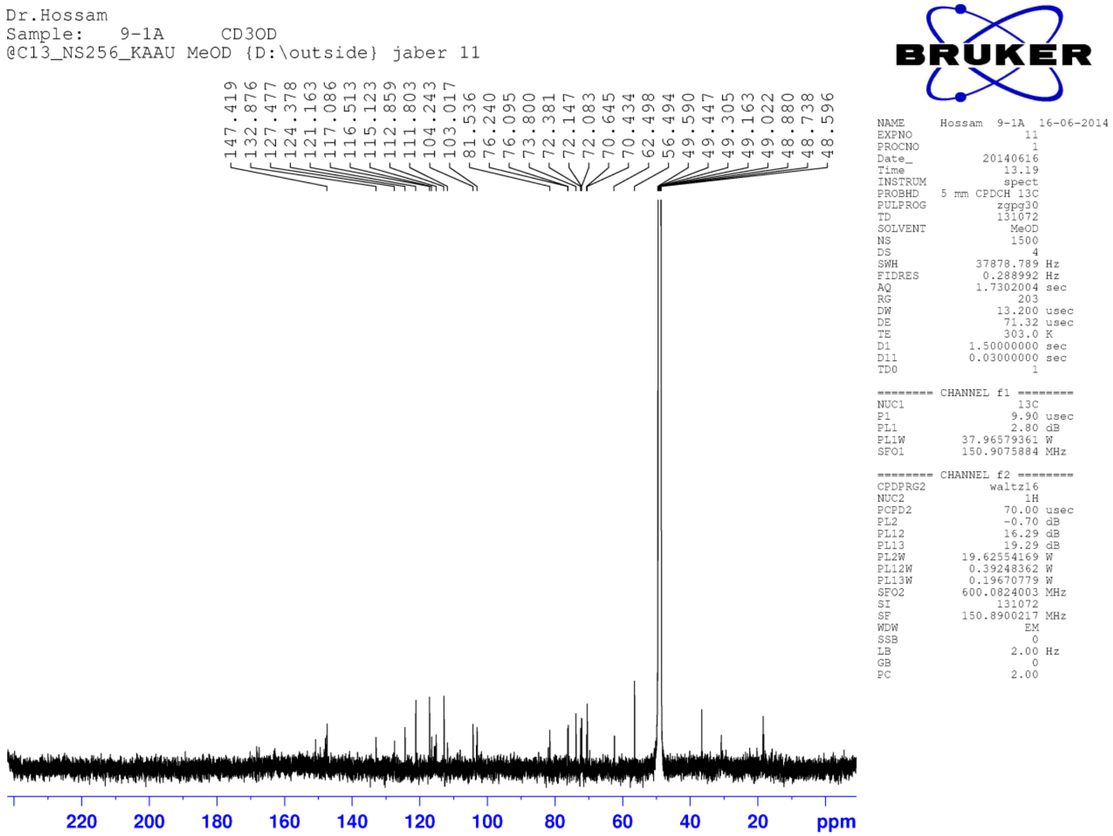

Supplementary Figure 6: ^{13}CNMR of compound 3

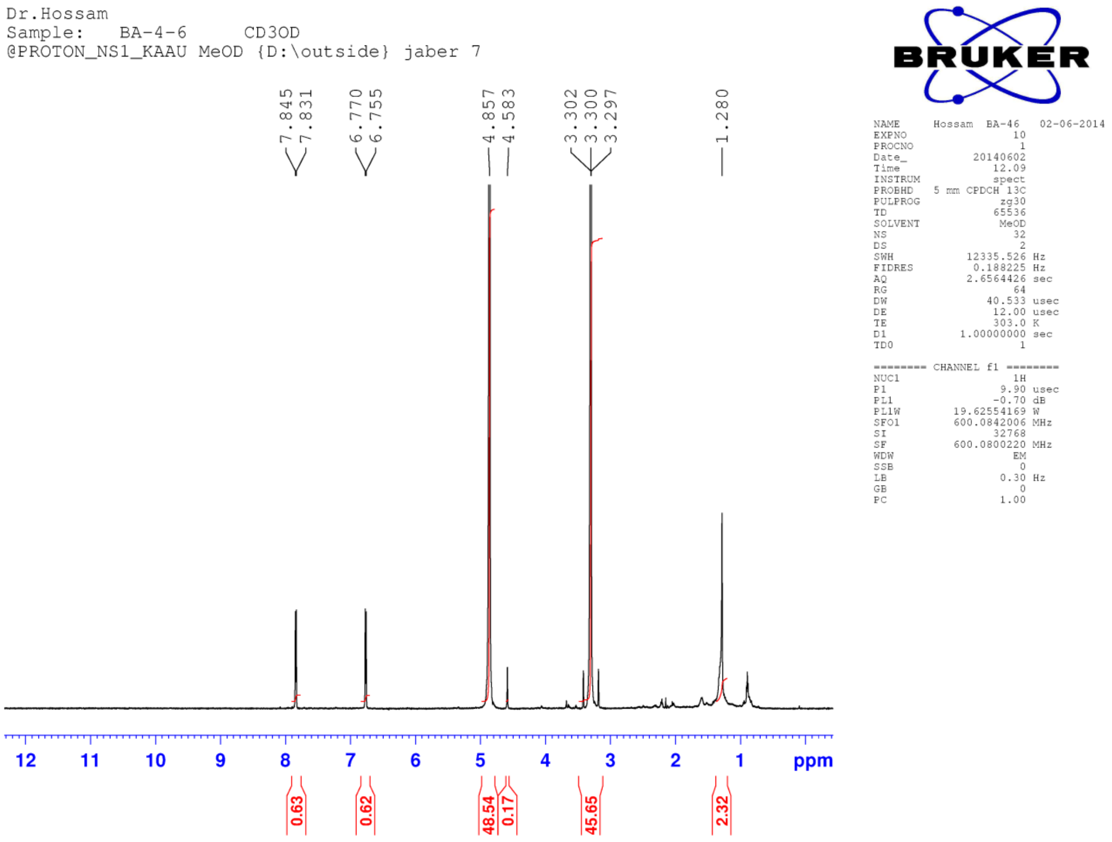

Supplementary Figure 7: HNMR of compound 4

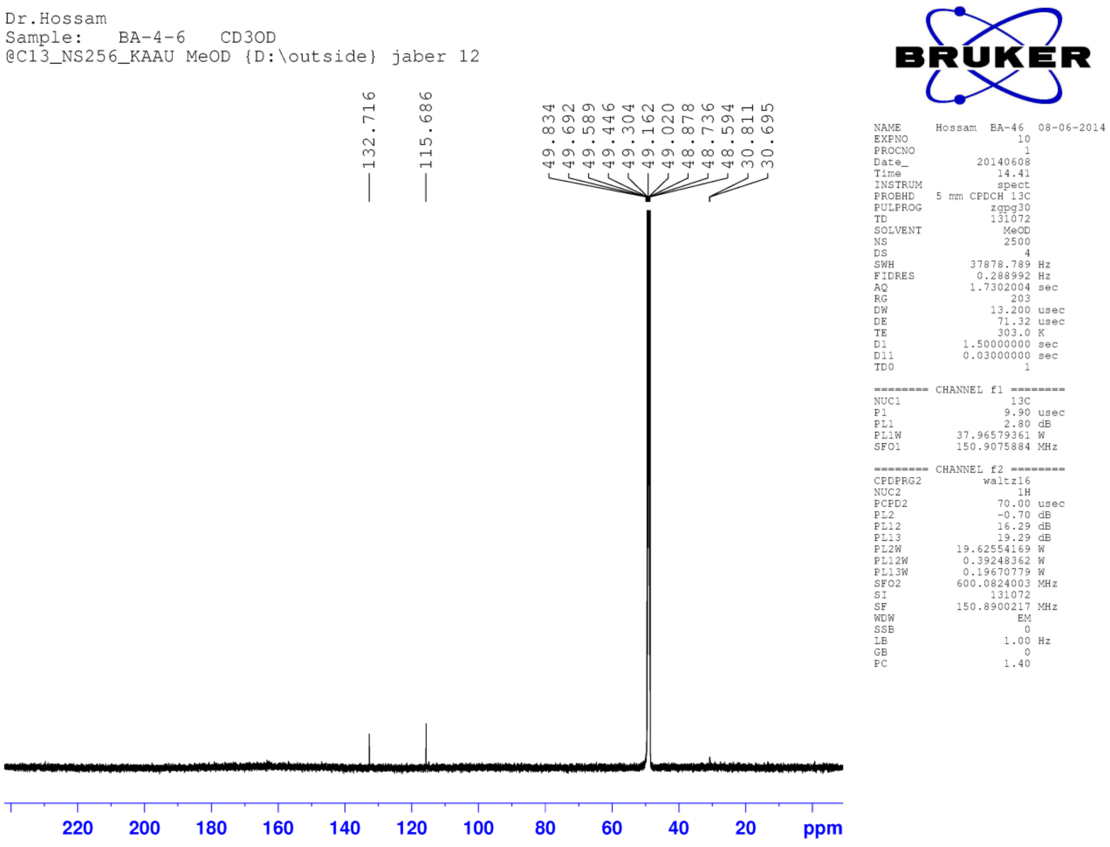

Supplementary Figure 8: ^{13}CNMR of compound 4

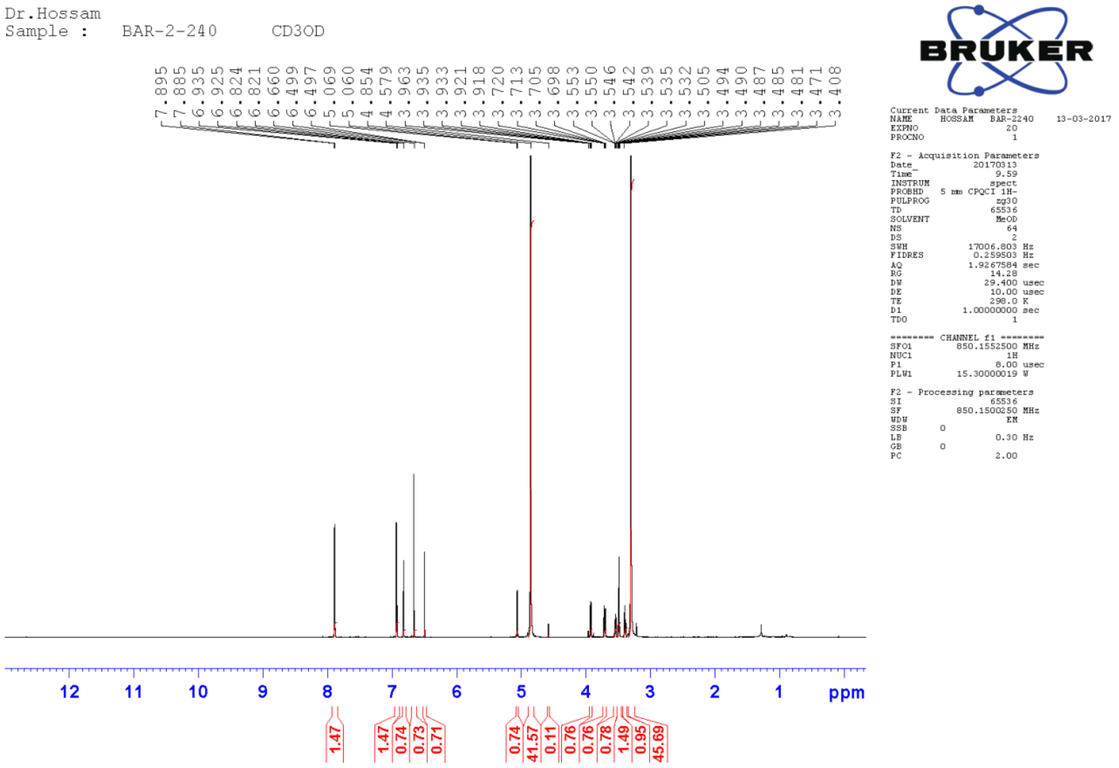

Supplementary Figure 9: HNMR of compound 5

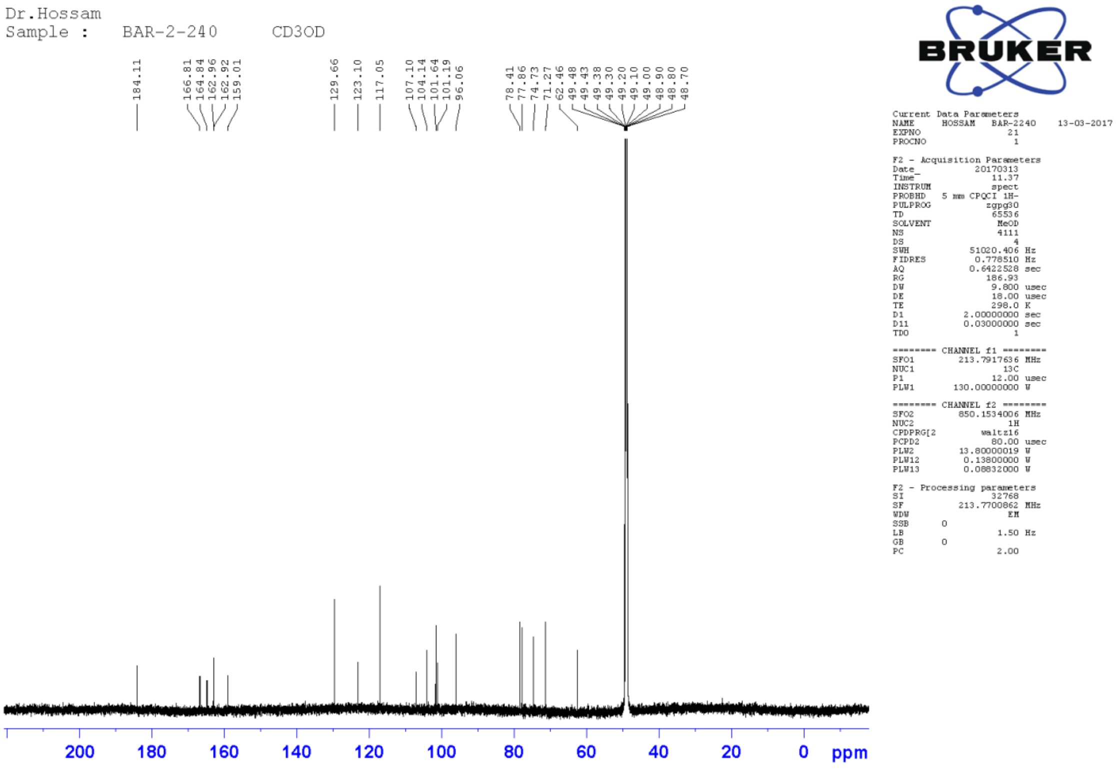

Supplementary Figure 10: ^{13}CNMR of compound 5
